# *GCK* exonic mutations induce abnormal biochemical activities and result in GCK-MODY

**DOI:** 10.3389/fgene.2023.1120153

**Published:** 2023-04-04

**Authors:** Tongtong Dai, Yun Yang, Juanjuan Zhang, Xiaoyu Ma, Lifen Chen, Caiping Zhang, Sheng Lv, Lin Li, Renqiao Tang, Ni Zhen, Wenli Lu, Chuanyin Li, Ronggui Hu, Yuan Xiao, Zhiya Dong

**Affiliations:** ^1^ Department of Pediatrics, Ruijin Hospital, School of Medicine, Shanghai Jiao Tong University, Shanghai, China; ^2^ School of Medicine, Guizhou University, Guiyang, China; ^3^ Cancer Center, Shanghai Tenth People’s Hospital, School of Medicine, Tongji University, Shanghai, China; ^4^ State Key Laboratory of Molecular Biology, Center for Excellence in Molecular Cell Science, Shanghai Institute of Biochemistry and Cell Biology, Chinese Academy of Sciences, Shanghai, China

**Keywords:** glucokinase, GCK, gene variants, biochemical activity, GCK-MODY

## Abstract

**Objective:** Glucokinase-maturity-onset diabetes of the young (GCK-MODY; MODY2) is a rare genetic disorder caused by mutations in the glucokinase (*GCK*) gene. It is often under- or misdiagnosed in clinical practice, but correct diagnosis can be facilitated by genetic testing. In this study, we examined the genes of three patients diagnosed with GCK-MODY and tested their biochemical properties, such as protein stability and half-life, to explore the function of the mutant proteins and identify the pathogenic mechanism of GCK-MODY.

**Methods:** Three patients with increased blood glucose levels were diagnosed with MODY2 according to the diagnostic guidelines of GCK-MODY proposed by the International Society for Pediatric and Adolescent Diabetes (ISPAD) in 2018. Next-generation sequencing (whole exome detection) was performed to detect gene mutations. The *GCK* gene and its mutations were introduced into the pCDNA3.0 and pGEX-4T-1 vectors. Following protein purification, enzyme activity assay, and protein immunoblotting, the enzyme activity of GCK was determined, along with the ubiquitination level of the mutant GCK protein.

**Results:** Genetic testing revealed three mutations in the *GCK* gene of the three patients, including c.574C>T (p.R192W), c.758G>A (p.C253Y), and c.794G>A (p.G265D). The biochemical characteristics of the protein encoded by wild-type *GCK* and mutant *GCK* were different, compared to wild-type GCK, the enzyme activity encoded by the mutant GCK was reduced, suggesting thermal instability of the mutant GST-GCK. The protein stability and expression levels of the mutant GCK were reduced, and the enzyme activity of GCK was negatively correlated with the levels of fasting blood glucose and HbA1c. In addition, ubiquitination of the mutant GCK protein was higher than that of the wild-type, suggesting a higher degradation rate of mutant GCK than WT-GCK.

**Conclusion:** *GCK* mutations lead to changes in the biochemical characteristics of its encoded proteins. The enzyme activities, protein expression, and protein stability of GCK may be reduced in patients with *GCK* gene mutations, which further causes glucose metabolism disorders and induces MODY2.

## 1 Introduction

Maturity-onset diabetes of the young (MODY) is a genetically heterogeneous group of monogenic endocrine disorders characterized by autosomal dominant inheritance and pancreatic beta cell dysfunction, accounting for 1%-2% of all diabetes patients (Ledermann, 1995). To date, 14 subtypes of MODY have been identified (Mccarthy and Hattersley, 2008), of which the subtype caused by glucokinase (GCK) is called GCK-MODY (MODY2) and accounts for 10%–60% of all MODY patients (Bishay and Greenfield, 2016). Most patients with GCK-MODY require only diet and exercise interventions, with no need for long-term follow-up and glucose-lowering therapy. Therefore, a correct diagnosis is important for the genetic counseling, treatment, and prognosis of patients with GCK-MODY.

However, GCK-MODY patients do not present the typical clinical symptoms of diabetes, rather only mild elevations in the levels of blood glucose and HbA1c (Steele et al., 2014; Chakera et al., 2015). According to the International Society for Child and Adolescent Diabetes (ISPAD) Clinical Practice Consensus Guidelines 2018 (Hattersley et al., 2018), the diagnosis of GCK-MODY should be suspected in cases with: 1) mild fasting hyperglycemia [fasting blood glucose (FBG) 5.5–8.0 mmol/L]; 2) mild elevation of HbA1c, but usually below 7.5%; 3) a small increment in blood glucose during an oral glucose tolerance test (OGTT; <3.5 mmol/L); 4) mild elevation of FBG in one of the parents, ranging from 5.5 to 8.5 mmol/L; 5) lack the characteristics of type 1 diabetes (no islet autoantibodies); 6) lack the characteristics of type 2 diabetes (marked obesity, acanthosis nigricans). In order to confirm the diagnosis, such cases should be tested for glucokinase gene mutations (GCK-MODY), which is the commonest cause of persistent, incidental hyperglycemia in the pediatric population.

GCK-MODY has an insidious onset, and most patients with GCK-MODY are diagnosed by chance. In addition, the clinical overlap between MODY and type 1 (T1D) or type 2 (T2D) diabetes makes accurate and timely diagnosis difficult. It is estimated that approximately 36% of GCK-MODY cases are misdiagnosed as T1D, and 51% are misdiagnosed as T2D, contributing to the incorrect treatment and management of GCK-MODY (Pihoker et al., 2013). Therefore, genetic testing is urgently required for the accurate diagnosis of GCK-MODY. A total of 620 mutations in the GCK gene have been described in 1,441 families (Osbak et al., 2009), which are relatively common in Caucasians but rare in Asians, with only 1% in the Hong Kong Chinese (Xu et al., 2005), 1% in the Japanese (Eto et al., 1993), and 2.5% in the Korean population (Hwang et al., 2006). However, the prevalence of GCK-MODY in Asians may be underestimated due to under- and misdiagnosis, and there are currently no available data for the Chinese Han population. In this study, three mutations in the GCK gene were identified in three proband patients with GCK-MODY from the Chinese Han population: c.574C>T (p.R192W), c.758G>A (p.C253Y), and c.794G>A (p.G265D). The biochemical properties of GCK, such as protein stability and half-life, were evaluated to explore the functions of the mutations, identify the pathogenic mechanisms of GCK-MODY, and analyze the correlation between the functional characteristics and clinical phenotypes of the three mutations.

## 2 Materials and methods

### 2.1 Patients

A total of three proband patients (one male and two females, aged 8–13 years) were included in this study. In the pedigree of the family in this study, elevated blood glucose levels were identified in at least two generations. The mother of patient 1 was in the diabetic stage, whereas the fathers of proband 2 and proband 3 were both in a state of pre-diabetes. The elevated blood glucose of the three patients was discovered by accident, and the patients presented with no typical symptoms of diabetes. No acute complications of diabetes, such as ketoacidosis, were reported. Laboratory tests showed that two patients (probands 1 and 2) were in the diabetic stage, whereas one patient (proband 3) only had impaired FBG, with a normal basal C-peptide level (normal range: 1.1–4.41 μg/L), a slightly higher HbA1c level (normal range: 6.4%–6.7%), as well as negative results for anti-glutamic acid decarboxylase autoantibodies (GADA), islet cell antibodies (ICA), and insulin autoantibodies (IAA; Table 1).

**TABLE 1:** Clinical characteristics of MODY2 probands in this study.

	Age at diagnosis	Age at present	Gender	BMI(kg/m^2^) at diagnosis	Glucose (mmol/L)		Insulin (μU/mL)		C-Peptide (μg/L)		ICA、IAA、GADA	HbA1c (%)	Glucose (mmol/L) of the carrier parent	
					0 h	2 h	0 h	2 h	0 h	2 h			0 h	2 h
Proband1	13	14	Male	16.72	8.96	11.10	15.51	105.4	2.35	9.1	Negative	6.7	7.32	12.50
Proband2	8	12	Female	17.55	6.34	13.31	15.84	122.5	2.89	13.75	Negative	6.4	6.74	11.58
Proband3	9	10	Female	16.43	6.42	6.97	7.58	19.97	1.34	4.10	Negative	6.6	6.58	7.42

BMI, body mass index; FBG, fasting blood glucose; ICA, islet cell antibody; IAA, insulin autoantibodies; GADA, glutamic decarboxylase autoantibody.

Based on the clinical characteristics and results of ancillary examinations, the diagnoses of T1D or T2D were excluded. These patients were initially diagnosed with MODY. According to the diagnostic guidelines of GCK-MODY by the ISPAD in 2018, the three patients were suspected of having GCK-MODY. Genetic testing was performed on the three patients and their parents to achieve an accurate diagnosis. This study was approved by the Ethics Committee of Ruijin Hospital, Shanghai Jiao Tong University, and written informed consent was obtained prior to the study.

### 2.2 Mutation screening

Blood samples were collected from the probands and their parents. Genomic DNA was extracted using a blood genomic DNA extraction kit according to the manufacturer’s instructions (Tiangen Biochemical Science and Technology, Beijing, China). Library preparation and exome capture were performed using the Hieff NGS^®^ OnePot DNA Library Prep Kit for Illumina^®^ (Yeasen Biotechnology, Shanghai, China) and a capture probe from the Twist Custom Panel (Twist Bioscience, CA, United States). High-throughput sequencing (HTS) of the libraries was performed in PE100 mode on the DNBSEQ-T7 platform (UWIC). Sequence data were quality checked using FASTQC software (v0.11.8). The reads were then aligned to the reference genome (GRCh37/hg19) using the BWA software. Finally, mutations were confirmed by Sanger sequencing (Weyhams Bio; forward primer: GGC​ATC​CTT​ACA​GCT​GCT​GA, reverse primer: TGC​CAG​AAA​ACA​AGG​GTC​GT). The pathogenicity of the mutations was assessed according to the American College of Medical Genetics and Genomics (ACMG) (Tavtigian et al., 2020).

### 2.3 Plasmid construction

The cDNA containing GCK, purchased from Sino Biological (HG29715-UT, Beijing, China), was used as a template and inserted into the 5′flag-tagged pCDNA3.0 and pGEX-4T-1 vectors. Mutations in GCK were introduced using targeted mutagenesis. The primers used are listed in Table 2. Positive clones were selected and sequenced to confirm the targeted mutations.

**TABLE 2:** Sequences of the primers used to construct the plasmids in this study.

Gene name	Forward/Reverse	Sequence (5′-3′)
GCK(p.G265D)	Forward	GGG​CGC​CTT​CGG​GGA​CTC​CGA​CGA​GCT​GGA​CGA​GTT​CCT​GC
GCK(p.G265D)	Reverse	GCA​GGA​ACT​CGT​CCA​GCT​CGT​CGG​AGT​CCC​CGA​AGG​CGC​CC
GCK(p.R192W)	Forward	GCT​TCT​GCG​AGA​CGC​TAT​CAA​ATG​GAG​AGG​GGA​CTT​TGA​AAT​GGA​TG
GCK(p.R192W)	Reverse	CAT​CCA​TTT​CAA​AGT​CCC​CTC​TCC​ATT​TGA​TAG​CGT​CTC​GCA​GAA​GC
GCK(p.C253Y)	Forward	GGG​GGA​CGA​GGG​CCG​CAT​GTA​CGT​CAA​TAC​CGA​GTG​GGG​CG
GCK(p.C253Y)	Reverse	CGC​CCC​ACT​CGG​TAT​TGA​CGT​ACA​TGC​GGC​CCT​CGT​CCC​CC
4T-1-GCK	Forward	AAT​CGG​ATC​TGG​TTC​CGC​GTA​TGG​CGA​TGG​ATG​TCA​CAA​GG
4T-1-GCK	Reverse	AGT​CAG​TCA​CGA​TGC​GGC​CGT​CAC​TGG​CCC​AGC​ATA​CAG​G

### 2.4 Cell culture and transfection

The human HEK293T cell line was kindly provided by Prof. Ronggui Hu (Chinese Academy of Sciences, Shanghai, China) and cultured in Dulbecco’s modified Eagle’s medium (DMEM, Life Technologies) supplemented with 10% fetal bovine serum (FBS), 100 U/mL penicillin, and 100 mg/mL streptomycin (both from Gibco, Layola) at 37°C in a humidified atmosphere containing 5% CO2. The plasmids were transfected into HEK293T cells using Lipofectamine 8,000 (Life Technologies, Carlsbad, CA, United States) according to the manufacturer’s instructions.

### 2.5 Immunoblotting

Cell lysates were subjected to sodium dodecyl sulfate-polyacrylamide gel electrophoresis (SDS-PAGE) and transferred to polyvinylidene fluoride (PVDF) membranes (Bio-Rad). Blots were incubated with antibodies against glyceraldehyde-3-phosphate dehydrogenase (GAPDH; 1:5000, 60,004-1-Ig, Proteintech, China), Flag (1:1000, 20543-1-AP, Proteintech Group, Chicago, United States), and ubiquitin (UB; 1:2000, 3936, Cell Signaling Technology, Danvers, MA). Secondary antibodies were labeled with horseradish peroxidase (HRP) and the signals were visualized using a Tanon 5,200 Imaging System (Tanon, Shanghai, China).

### 2.6 Induced expression of recombinant protein

An overnight culture of *E. coli* BL21(DE3)harboring the recombinant plasmid Pgex-4T-1/GCK was inoculated in the Luria-Bertani medium. The cultures were incubated at 37°C with shaking. When the culture reached an optical density of 0.5 at 600 nm, isopropyl-beta-D-1-thiogalactoside (IPTG) was added to induce gene expression. The bacteria were harvested and sonicated on ice in a lysis buffer. GST-GCKs were produced in *E. coli* and then purified from crude extracts as described (Liang et al., 1995). The purity of the recombinant protein was checked on a 10% precast SDS-PAGE gel stained with Coomassie Brilliant Blue (CBB). The protein band at 78 kDa represents the glucokinase (GCK)-GST fusion protein.

### 2.7 Analysis of mutant and wild-type GST-GCK fusion protein enzymatic activity

The mutant and wild-type (WT) GST-GCK fusion proteins levels were measured and adjusted to the same concentration using a BCA protein concentration kit (P0010S, Beyotime, China), and were further confirmed by the Bradford method. The GCK activity of GST protein and mutant and WT GST-GCK fusion proteins was assayed spectrophotometrically using the hexokinase activity assay kit (AKSU061M, boxbio, China) after incubation in a water bath at 25°C, 37°C, 42°C and 50°C for 30 min (Kesavan et al., 1997), respectively, where the GST protein was used as a negative control.

### 2.8 Analysis of protein stability

HEK293T cells were inoculated into 6-well plates (1.2 × 106 cells/well) and cultured overnight. Then, empty and recombinant plasmids constructed with the pCDNA3.0 vector (mutant and WT GCK) were transiently transfected into the HEK293T cells. Each experiment was repeated three times. After 24 h of transfection, the solution was replaced with fresh and complete medium, and Cycloheximide (CHX) was added, followed by incubation for 0, 2 and 4 h, respectively. Finally, the cells were harvested and lysed with 6×SDS to determine the degradation rate of GCK and its mutants using immunoblotting.

### 2.9 Detection of ubiquitination

*In vivo* ubiquitination assays were performed as previously described (Liu et al., 2014). HEK293T cells were transfected with the indicated plasmids for 48 h and immunoprecipitated with anti-FLAG beads. After rotational incubation, cells were lysed with ristocetin-induced platelet aggregation (RIPA) lysis buffer [50 mM Tris-Cl (pH 7.4), 150 mM NaCl, 5 mM EDTA, 1% (v/v) Triton X-100, 0.5% sodium pyrophosphate, 0.1% SDS, and protease inhibitor cocktail (Bimake, B14001, Houston, TX, United States)] and boiled for 10 min in SDS-PAGE sample buffer, followed by immunoblotting with the indicated UB antibodies (1:2000, 3936, Cell Signaling Technology, Danvers, MA).

### 2.10 Statistical analysis

Results are presented as the mean ± standard deviation (SD). GraphPad Prism 8.0 (San Diego, CA, United States) and one-way analysis of variance (ANOVA) were used to analyze the statistical differences between groups. Statistical significance was set at *p* < 0.05.

## 3 Results

### 3.1 Results of genetic testing

**FIGURE 1:**
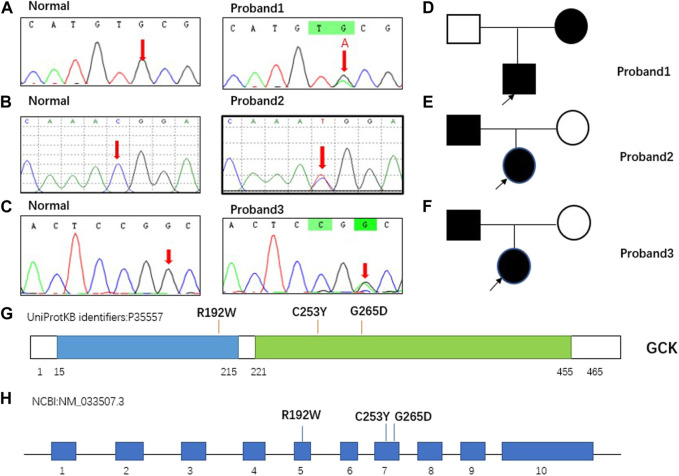
Pedigree of MODY2 family in this study and mutations carried by proband. **(A)**. GCKc.758G > A (p. C253Y) carried by proband 1 confirmed by Sanger sequencing. The normal site was only the base G, whereas the same site in proband 1 was G and A. **(B)**. GCK c.574C > T (p. R192W) carried by proband 2 was confirmed by Sanger sequencing. The normal site was only base C, whereas the same site in proband 2 was C and T. **(C)**. GCK c.794G > A (p. G265D) carried by proband 3 was confirmed by Sanger sequencing. The normal site was only base G, whereas the same site in proband 3 was G and A. **(D–F)**. Squares represent males and circles represent females. Filled black symbols indicate members carrying mutations and having clinical symptoms, and empty symbols indicate family members without mutations. The proband is marked with a black arrow. **(G–H)**. Schematic view of human GCK protein and gene showing the location of the three variants involved in our study. GCK is a 465-residue, 52-kDa enzyme comprising two domains, hereafter referred to as the large domain (green) and the small domain (blue). The overall structure of the protein was comprised of a large and a small globular domain connected by a hinge made up of three flexible loops. The binding site for the activator was localized in the hinge region. The mutations identified in this study were located in two regions. GCK contains 10 exons, and the three mutations detected in this study were located in exons 5 and 7.

*GCK* gene mutations were discovered in all three proband patients, namely c.758G > A (p.C253Y), c.574C > T (p.R192W), and c.794G > A (p.G265D), all of which were heterozygous mutations, with two (proband 2 and 3) derived from the father and one (proband 1) from the mother. According to the ACMG guidelines, the variants of probands 1 and 2 were classified as “P”-pathogenic, and proband 3 as “LP”- likely pathogenic. The mutation in proband 3 has not been previously reported. The *GCK* gene mutations and classification based on the ACMG guidelines are shown in Table 3 and the specific classification reasons can be seen in Supplementary Tables S1–S3. The pedigree of the MODY2 family, Sanger sequencing, structural domains of GCK, and mutation sites are shown in Figure 1. A literature review found no reports on the functional characteristics of the three mutations. To investigate the relevant pathogenic mechanisms, we conducted a functional study of the three mutations in the *GCK* gene.

**TABLE 3:** Genotype and variant classification of the patients in this study.

No.	Exon	cDNA	Protein	Genotype	Origin	ACGM
1	7	c.758G>A	p.C253Y	Heterozygous	Mother	P/PM1+PM2+PM5+PP3
2	5	c.574C>T	p.R192W	Heterozygous	Father	P/PM1+PM2+PM5+PP3+PP5
3	7	c.794G>A	p.G265D	Heterozygous	Father	LP/PM1+PM5+PP3+PM2

P, pathogenic; LP, likely pathogenic. PM1: Located in a mutational hot spot and/or critical and well-established functional domain (e.g., active site of an enzyme) without benign variation. PM2: Absent from controls (or at extremely low frequency if recessive) in Exome Sequencing Project, 1000 Genomes Project, or Exome Aggregation Consortium. (Pathogenic, Moderate). PM5: Novel missense change at an amino acid residue where a different missense change determined to be pathogenic has been seen before. (Pathogenic, Moderate): PP3: Multiple lines of computational evidence support a deleterious effect on the gene or gene product (conservation, evolutionary, splicing impact, etc.). PP5: Reputable source recently reports variant as pathogenic, but the evidence is not available to the laboratory to perform an independent evaluation. (Pathogenic, Supporting).

### 3.2 Production of recombinant mutant and WT GST-GCK

**FIGURE 2:**
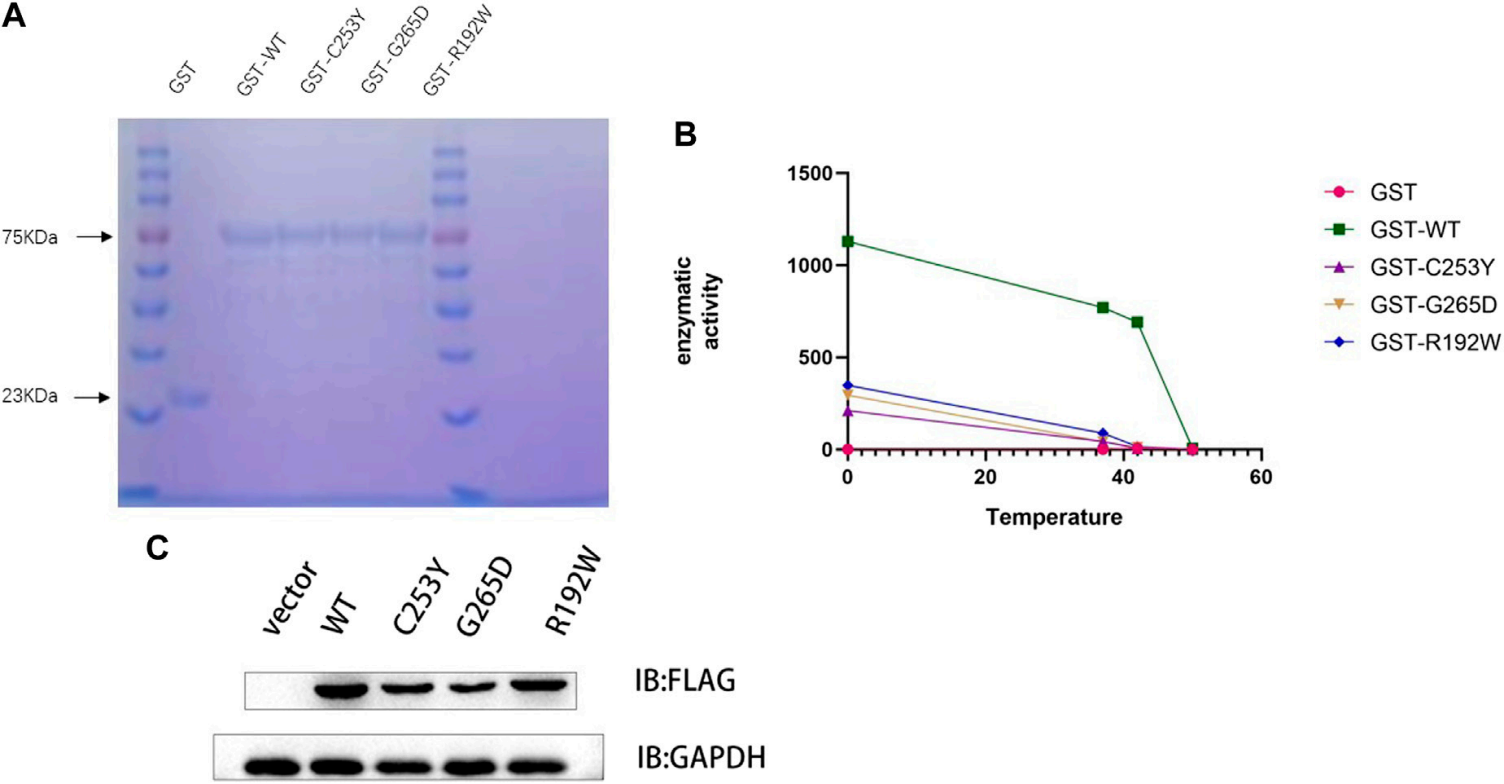
Mutation of the GCK gene reduces enzyme activity and protein expression. **(A)**. Protein content was detected using the Coomassie Brilliant Blue method. The same volume (10 μL) of protein solution was added to each loading well, and the same band width indicated the same protein concentration. The GST protein (23 kDa) was used as a control. **(B)**. The enzyme activity was detected after the protein was heated in a water bath at 37°C, 42°C, and 50°C for 30 min. The graph shows that the enzyme activity of the wild-type protein was much higher than that of the mutant protein at the same temperature. At 42°C, the wild-type protein still contained at least 50% enzyme activity, whereas the mutant protein lost its activity. **(C)**. Detection of protein levels in WT GCK and its mutants by immunoblotting. Empty vector or FLAG-tagged wild-type GCK/GCK (c.758G>A)/GCK (c.794G>A)/GCK (c.574C>T) were transfected into HEK293T cells, and the protein levels of FLAG-tagged protein and GAPDH were detected by immunoblotting. The expression of the wild-type protein was higher than that of the mutant protein. We detected the gray scale of the bands by ImageJ and used ANOVA test to verify whether there were differences in protein expression. P‹0.05, significant different.

To investigate the activities of GCK, the WT and mutant GCK were expressed as GST-GCK fusion proteins in a bacterial expression system (Shen et al., 2011). The protein was separated by SDS-PAGE and a single 78 kDa band was obtained (GCK protein, 52 kDa; GST protein, 26 kDa). The protein concentration was determined by the BCA method (Figure 2A), and the results showed that the concentrations of WT protein, R192W, C253Y, and G265D were 56 g/L, 48 g/L, 43 g/L and 42 g/L respectively. The production of the mutant fusion protein was lower than that of the WT. The production of the mutant fusion proteins varied as follows: R129W > C253Y > G265D.

### 3.3 Enzyme activity and thermal stability of recombinant WT and mutant GST-GCK

The enzyme activities of WT and mutant GST-GCK were tested at different temperatures (Figure 2B), and the results showed that the enzyme activity of WT GST-GCK was higher than that of the mutant GST-GCK at any temperature. The mutant GST-GCK enzyme activities were ranked as follows: c.574C>T (p.R192W) > c.758G>A (p.C253Y) > c.794G>A (p.G265D). After incubation at 40°C for 30 min, the enzyme activity of WT GST-GCK remained high, whereas the mutant GST-GCK was inactivated, suggesting the thermal instability of the mutant GST-GCK.

### 3.4 Expression of recombinant mutant and WT GCK *in vivo*

To investigate the expression of mutant and WT GCK *in vivo*, empty vector or FLAG-tagged WT GCK/GCK (c.758G>A)/GCK (c.794G>A)/GCK (c.574C>T) was transfected into HEK293T cells. The protein levels of FLAG-tagged protein and GAPDH were detected by immunoblotting, and the results showed that the expression level of WT GCK was significantly higher than that of the mutant GCK (Figure 2C).

### 3.5 Protein stability and ubiquitination levels of recombinant mutant and WT GCK

**FIGURE 3:**
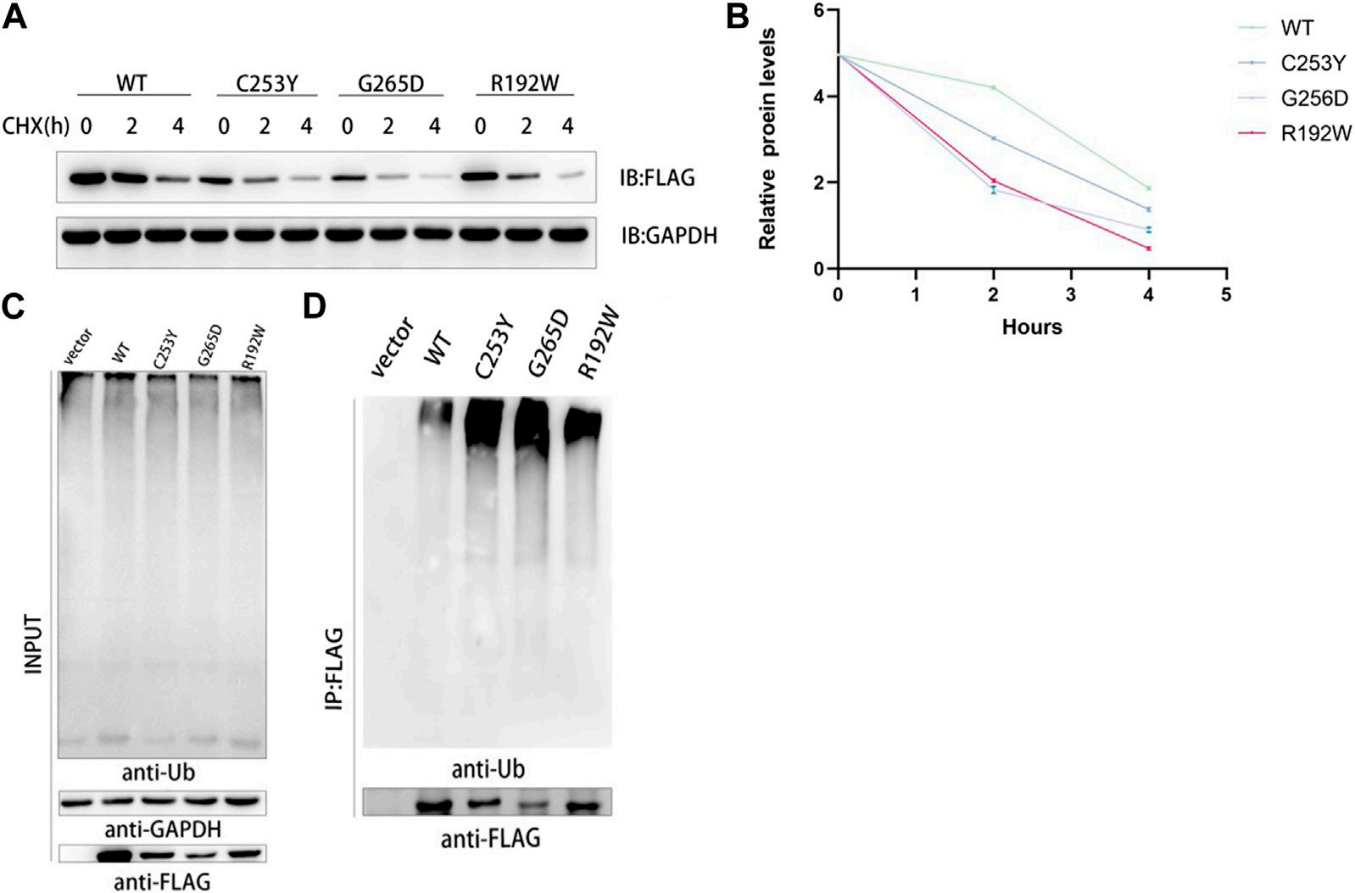
Analysis of the protein stability of wild type and mutant GCK in mammalian cells. **(A)**. HEK293T cells transfected with plasmids containing GCK-WT or its mutants were treated with CHX (1 μg/μL) for 0, 2 and 4 h. Total cell lysates were analyzed for GCK protein levels using western blotting. **(B)**. ImageJ was used to detect the gray levels of wild-type and mutant GCK proteins at 0 h and to homogenize them to better compare the degradation rates of proteins. The results were calculated from three independent experiments. Each bar represents the mean ± SD. **(C-D)**. The ubiquitination level of FLAG-GCK with the C253Y/G265D/R192W mutation was higher than that of the WT FLAG-tagged protein. Ubiquitination of the wild-type GCK protein was less than that of the mutants. FLAG-tagged wild-type or mutant GCK were expressed in HEK293T cells and immunoprecipitated using anti-FLAG beads, followed by immunoblotting with anti-Ub to detect ubiquitination signals.

CHX prevents the translocation of tRNA during translation by binding to the 80S ribosome, thereby inhibiting the synthesis of new proteins (Nakashima et al., 1981; Koga et al., 2021). The stability of the synthesized proteins was determined based on the protein levels of FLAG-tagged proteins and GAPDH using western blotting, and the results showed that the protein content of WT GCK *in vitro* was higher than that of the mutant GCK after incubation with CHX for 4 h, indicating that the half-life of WT GCK was longer than that of mutant GCK; thus, the protein stability of the wild-type GCK was higher than that of the mutant GCK (Figures 3A, B). In this study, GCK and its mutant proteins were used as substrates for ubiquitination to explore the differences between WT and mutant GCK. The results showed that the ubiquitination level of mutant GCK was higher than that of WT GCK, suggesting a higher degradation rate in mutant GCK than in WT GCK (Figures 3C, D).

## 4 Discussion

GCK-MODY is a specific type of diabetes mellitus characterized by autosomal dominant inheritance, which usually occurs before 25 years of age and exhibits a defect in glucose-stimulated insulin secretion. GCK-MODY does not require specific treatment, except in pregnant women (American Diabetes Association, 2020). However, it is often misdiagnosed as T1DM or T2DM in clinical practice, and genetic testing can facilitate accurate diagnosis and reduce unnecessary treatments. Our study focused on the correlation between pathogenic mechanisms, functional characteristics, and clinical phenotypes in three patients with GCK-MODY.

The clinical manifestations of the patients in this study met the diagnostic guidelines for GCK-MODY proposed by the ISPAD in 2018. Three mutations were identified in these patients and their families through genetic testing: c.574C>T (p.R192W), c.758G>A (p.C253Y), and c.794G>A (p.G265). The c.574C>T (p.R192W) mutation was located in exon 5 and the other two were located in exon 7. Exons 5 and 7 were common mutation sites in 110 Asian patients with GCK-MODY based on a literature search in PubMed, Embase, MEDLINE, Web of Science, CNKI, and Wanfang (Zhou et al., 2020), suggesting that the hotspot mutation sites of GCK in Asian patients might be located in exons 5 and 7, whereas the hotspot mutation sites of GCK in Europe patients might be located in exons 7 and 10 (Pruhova et al., 2010).

GCK is a glycolytic enzyme mainly found in pancreatic β-cells and hepatocytes (Rexford et al., 2017). It catalyzes the ATP-dependent phosphorylation of glucose to produce glucose-6-phosphate, which is the first step in glucose metabolism. GCK plays a critical role in the release of insulin as a key regulatory enzyme and glucose sensor in pancreatic β-cells (Kim et al., 2008). When the blood glucose level increases, GCK activity in pancreatic β-cells is enhanced to promote glucose metabolism, thereby lowering the blood glucose level. In early studies, Byrne pointed out that in patients with GCK-MODY, the glucose sensitivity of pancreatic β-cells was reduced, and the threshold for insulin secretion was increased due to the decrease in GCK activity induced by heterozygous mutations in the GCK gene; thus, patients with GCK-MODY showed a higher level of FBG compared to the controls (Byrne et al., 1994).

The regulatory mechanism of GCK activity related to GCK gene mutations has been investigated, and studies have shown that thermal instability, which is inversely proportional to the level of environmental glucose, can affect GCK activity *in vivo* (Kesavan et al., 1997; Miller et al., 1999; García-Herrero et al., 2007). In our study, the mutant GCK lost its activity above 40°C, whereas the WT GCK maintained its activity at 50°C. It seems that the mutant GCK cannot tolerate high temperatures, which may show the thermal instability. In addition, our results demonstrated that the activity of mutant GCK was lower than that of WT GCK regardless of the temperature, indicating that the mutations might directly result in a decrease in GCK activity. In our study, GCK activity at the same temperature ranked as follows: GCK-R192W > GCK- G265D > GCK- C253Y, which was consistent with the results of blood glucose monitoring in clinical practice; meanwhile, the levels of blood glucose and HbA1C in the three patients were as follows: proband 2 (GCK-R192W) < proband 3 (GCK-G265D) < proband 1 (GCK- C253Y). Based on the above findings, it can be inferred that GCK activity may be correlated with FBG and HbA1C levels. Notably, after incubating the WT and mutant GCK in a 37°C water bath (simulating normal body temperature in humans) for 30 min, the activity of mutant GCK was found to be much lower than that of WT GCK, suggesting that a decrease in GCK activity *in vivo* might result in the disruption of the phosphorylation of glucose to glucose 6-phosphate process, and may result in glucose metabolism disorders.

In addition to thermal instability of GCK mutants, GCK activity may also be influenced by other mechanisms. Some studies have suggested that *GCK* mutations downregulate the production of GCK *in vitro* (Sagen et al., 2006), and one study found that the production of GCK mutants, such as G72R, A208T, and M210K, was significantly lower than that of WT GCK. In the present study, the c.574C>T (p.R192W), c.758G>A (p.C253Y), and c.794G>A (p.G265D) GCK mutants showed lower production *in vitro* than WT GCK. Simultaneously, cells transfected with the WT and mutant plasmids were incubated with CHX for 0, 2, and 4 h, and the results showed that the protein stability of the mutant GCK was lower than that of the WT GCK, and the ubiquitination levels of mutant GCK were higher than those of the WT GCK. Ubiquitination is an enzymatic process that involves the bonding of a Ub protein to a substrate protein and has the potential to regulate protein stability, function, and protein-protein interactions (Hoeller and Dikic, 2009). The ubiquitin-proteasome (UP) pathway is a common pathway of endogenous protein degradation, in which proteins are degraded by the proteasome after modification by ubiquitination. All the above findings indicate that compared to the WT GCK, the mutant GCK had a weaker protein stability and was more susceptible to degradation, thus presenting a shorter half-life. Therefore, it can be concluded that lower expression and faster degradation of the mutant GCK also contribute to elevated blood glucose levels in patients with GCK mutations. Notably, despite the longer half-life and higher expression level of C253Y compared to G265D, the levels of blood glucose and HbA1C were found to be higher in proband 1 (GCK- C253Y) than in proband 3 (GCK-G265D), suggesting that the activity of mutant GCK may play a more significant role in glucose metabolism than the production of mutant GCK.

Several other mechanisms may also be involved in the regulation of GCK activity. Studies have revealed that GCK consists of both large and small globular structural domains, connected by a hinge composed of three flexible loops. In the presence of glucose and activators, the space between the two structural domains resembles a narrow, deep cleft containing a glucose-binding pocket. The binding site for the activators is located in the hinge and not in the glucose-binding pocket (Kamata et al., 2004). GCK has two conformations, among which the super-open conformation represents a low glucose-affinity, non-catalytically active form of GCK, whereas the closed conformation corresponds to a high glucose-affinity form of GCK, and glucose and ATP are bound at the active site during catalysis. Glucose exerts its role by binding to the glucose-binding pocket and inducing a change in the conformation of GCK from the super-open form to the closed form, which is an extremely rapid and reversible process. In this study, all the mutations in the three patients were located in the two structural domains, with one in the small domain and two in the large domain. In this study, R192W, C253Y and G265D are located in close proximity to the glucose binding site, resulting in obstruction of the glucose binding pocket (Valentínová et al., 2012). We believe that enzyme activity of the mutant GCKs is reduced because they do not bind to glucose as effectively as WT-GCK, resulting in an increase in the patients’ blood glucose (Zelent et al., 2008). Thus, X-ray diffraction (XRD) analysis is necessary in future studies to investigate impact of these mutations on the structure of GCK.

There are still some limitations in this study. We mainly studied the biochemical signature of proteins *in vitro*, while the human body is a dynamic environment in which many pathways and cells are in constant exchange and communication. Therefore, the difference between the biochemical functions of mutant protein and wild-type protein *in vivo* still needs to be further studied. In order to make the link between genetic mutations and the clinical phenotype of diabetes more accurate, the participation of genetically mutated mice may be required. Moreover, we tried to use the result that the mutant GST-GCK cannot tolerate high temperature to represent their poor thermal stability. In order to verify the thermal stability of the mutant and WT GCK, the melting temperature should be test. In conclusion, we investigated the changes in the functional characteristics of three GCK mutants, c.574C>T (p.R192W), c.758G>A (p.C253Y), and c.794G>A (p.G265D), and explored the correlation between functional characteristics and clinical phenotypes. The results showed that reduced activity, decreased production, and protein instability resulting from *GCK* gene mutations may be the main causes of hyperglycemia in MODY-GCK patients.

## Data Availability

The data presented in the study are deposited in the China National GeneBank (CNGB) Nucleotide Sequence Archive (CNSA: https://db.cngb.org/search/project/CNP0003798/) repository, accession number CNP0003798.
