# Supplementary material for: GCK exonic mutations induce abnormal biochemical activities and result in GCK-MODY
Source: Front Genet. 2023 Apr 4;14:1120153. doi: 10.3389/fgene.2023.1120153 (PMC10110986; doi:10.3389/fgene.2023.1120153)
Supplement: Supplementary file 1 [file DataSheet1.docx]

Supplementary Material

*GCK exonic mutations induce abnormal biochemical activities and result in GCK-MODY*

**Supplementary Table 1. ACMG classification of proband 1（c.758G＞A,p.C253Y）**

| **Classification** | **Explains** |
| --- | --- |
| PM1 | Located in the hot-spot of length 17 amino-acids has 35 missense/in-frame variants (15 pathogenic variants, 23 uncertain variants and no benign), which qualifies as strong pathogenic. |
| PM2 | Variant not found in gnomAD genomes, good gnomAD genomes coverage = 32.2. Variant not found in gnomAD exomes, gnomAD exomes coverage is unavailable. |
| PM5 | p.C253G is classified Likely Pathogenic by UniProt Variants  (and confirmed using ACMG) |
| PP3 | MetaRNN = 0.985 is greater than 0.939 →strong pathogenic |

**Supplementary Table 2. ACMG classification of proband 2(c.574C>T,p.R192W)**

| **Classification** | **Explains** |
| --- | --- |
| PM1 | Located in the hot-spot of length 17 amino-acids has 16 missense/in-frame variants (8 pathogenic variants, 8 uncertain variants and no benign), which qualifies as moderate pathogenic. |
| PM2 | Variant not found in gnomAD genomes, good gnomAD genomes coverage. = 31.8. GnomAD exomes homozygous allele count = 0 is less than 2 for AD/AR gene GCK, gnomAD exomes coverage is unavailable. |
| PM5 | p.R192Q is classified Likely Pathogenic by the VarSome community in article 34373539 (and confirmed using ACMG). |
| PP3 | MetaRNN = 0.951 is greater than 0.939 → strong pathogenic. |
| PP5 | ClinVar classifies this variant as Pathogenic, 2 stars (multiple consistent, reviewed Dec '22, 8 submissions), citing 11 articles (36257325, 34393998, 32741144, 31576961, 30656436 and 6 more). UniProt Variants classifies this variant as Pathogenic, citing 2 articles (17573900 and 16965331). |

**Supplementary Table 3. ACMG classification of proband3(c.794G＞A，p.G265D)**

| **Classification** | **Explains** |
| --- | --- |
| PM1 | Hot-spot of length 17 amino-acids has 26 missense/in-frame variants (11 pathogenic variants, 15 uncertain variants and no benign), which qualifies as moderate pathogenic. |
| PM2 | Variant not found in gnomAD genomes, good gnomAD genomes coverage = 32.2. Variant not found in gnomAD exomes, gnomAD exomes coverage is unavailable. |
| PM5 | p.G265S is classified Likely Pathogenic by the VarSome community in article 22820548 (and confirmed using ACMG). |
| PP3 | MetaRNN = 0.892 is between 0.841 and 0.939 →moderate pathogenic. |
